# Multifunctional Eco-Friendly Synthesis of ZnO Nanoparticles in Biomedical Applications

**DOI:** 10.3390/molecules27020579

**Published:** 2022-01-17

**Authors:** Amal Mohamed Al-Mohaimeed, Wedad Altuhami Al-Onazi, Maha Farouk El-Tohamy

**Affiliations:** Department of Chemistry, College of Science, King Saud University, P.O. Box 22452, Riyadh 11495, Saudi Arabia; woalonazi@ksu.edu.sa (W.A.A.-O.); moraby@ksu.edu.sa (M.F.E.-T.)

**Keywords:** oat extract, zinc oxide nanoparticles, green synthesis, antibacterial, antioxidant, pharmaceutical analysis

## Abstract

This work describes an environmental-friendly preparation of ZnO nanoparticles using aqueous oat extract. The advanced electrochemical and optical features of green synthesized ZnONPs displayed excellent antibacterial activity and exhibited an important role in pharmaceutical determinations. The formation of nanoscale ZnO was confirmed using various spectroscopic and microscopic investigations. The formed nanoparticles were found to be around 100 nm. The as-prepared ZnONPs were monitored for their antibacterial potential against different bacterial strains. The inhibition zones for ZnONPs were found as *Escherichia coli* (16 mm), *Pseudomonas aeruginosa* (17 mm), *Staphylococcus aureus* (12 mm) and *Bacillus subtilis* (11 mm) using a 30-µg mL^−1^ sample concentration. In addition, ZnONPs exhibited significant antioxidant effects, from 58 to 67%, with an average IC_50_ value of 0.88 ± 0.03 scavenging activity and from 53 to 71% (IC_50_ value of 0.73 ± 0.05) versus the scavenging free radicals DPPH and ABTS, respectively. The photocatalytic potential of ZnONPs for Rhodamine B dye degradation under UV irradiation was calculated. The photodegradation process was carried out as a function of time-dependent and complete degradation (nearly 98%), with color removal after 120 min. Conclusively, the synthesized ZnONPs using oat biomass might provide a great promise in the future for biomedical applications.

## 1. Introduction

Nanoscale materials, especially metal oxide nanostructures, are known as a distinctive group of nanopaerticles with unique physicochemical properties and possess broad applications in various scientific areas such as sensing technology [1,2], cell adhesion and tissue engineering [3], industrial packaging [4,5] catalysis [6,7] and biomedical investigations [8,9].

Zinc oxide nanoparticles (ZnONPs) have gained extensive awareness for their advanced biocompatible features and high strength under utmost circumstances [10,11]. ZnONPs have been used in medicine [12], sensors [13], industrial additives [14], photocatalysis [15], antioxidants and antibacterial potentials [16,17]. The increased adaptation of pathogens and microorganisms’ resistance to antibiotics requires the synthesis of new components that have the ability to destroy the microorganism without the probability of adaptations appearing. Thus, the production of new components that possesses antimicrobial properties is greatly necessary [18]. Zinc oxide is recorded as safe metal oxide (21CFR182.8991) by the United States Food and Drug Administration. It is commonly used as a food preservative in the fortification of cereal-based foods. Because of its antimicrobial potential, ZnO has also been used in the packaging of food cans such as meat, corn, fish and peas to prevent spoilage and preserve the color [19]. Recently, ZnONPs have gained more attention as an antimicrobial agent than bulk particles. ZnO nanoparticles have excellent interaction with microorganisms, due to their high ratio of surface area to volume and minimized size. Several studies have reported that ZnONPs possess selective toxicity to Gram-positive and Gram-negative bacteria such as *Escherichia coli*, *Salmonella* and *Staphylococcus aureus* but displayed minimal effect on human cells [20,21].

The conventional methods for the synthesis of ZnONPs need hazardous chemicals with a high quantity of extrinsic heat; further, they produce various harmful side by-products that could possess effective biological and environmental threats. Therefore, a considerable need to progress eco-friendly, compatible, profitable, energy-efficient, green synthesis methods to avoid the use of toxic materials in the synthesis of nanoparticles [22].

To defeat these drawbacks, natural substances (marine algae, bacteria and plants) display excellent potential materials for the green preparation of metallic oxide nanosized. The superiority of utilizing biomasses of plants in the preparation of ZnONPs is simple accessibility and the fact that they are eco-friendly and mostly safe [23]. These plants contain various types of biomolecules, such as phenolic compounds, terpenoids, acids and alkaloids, which can potentially act as reducing and stabilizing substances in the preparation of nanostructured metal oxides [24]. Oat (*Avena sativa*) is a kind of cereal grain belonging to the plant family *Poaceae grass*. The grain refers particularly to the edible seeds of oat grass, that is approved by the Food and Drug Administration as healthy food labels correlated to a decreased risk of various disorders, including cardiovascular disease and diabetes. Oat grains are also used in weight control and digestive health [25,26,27,28]. A literature survey claims that *A. sativa* extract contains macromolecules such as phenolic compounds, amino acids, carotenoids, proteins, starch and unsaturated fatty acids [29]. These biomolecules, in addition to the above-described medicinal properties, exhibit antimicrobial and antioxidant activities [30,31]. Besides, the phytochemical profile of oat biomass includes natural chemical components which have been employed for the biosynthesis of nanomaterials such as metal and metal oxide nanoparticles [32,33]. Additionally, one of the major environmental hazards is the effluent from the dyeing and pigment industries, which possess various toxic and non-biodegradable by-products [34]. A water-soluble xanthene dye (Rhodamine B (RB)) is one of the common dyes that are generally used in many industrial implementations, such as plastic dying and trace dying for determining the water rate flow and direction, as well as applications in the textile industry, pharmaceuticals and cosmetics. This dye is considered one of the most organic environmental pollutants [35].

The photodecomposition of these pollutants to more safe wastes can be considered as a potent method to minimize the vast quantity of waste materials produced by industry and liberated into surrounding environment [36]. Various conventional probes, including spectroscopy, chromatography and chemical oxidation, have been addressed in different industries for dye bleaching [37]. Additionally, a few recent techniques, including solar photo-Fenton, photobiological degradation and sonochemical degradation, have been reported for dye removal [38,39,40]. Nevertheless, these techniques possess certain downsides, such as high energy consumption, high costs and complicated instrumentation. In addition, they consume large amounts of toxic materials. Therefore, it is important to suggest a simpler, easy and less environmentally polluting probe to eliminate these toxic dyes from waste products. Recently, nanoscience research has been concerned with using green nanotechnology in the biosynthesis and characterization of metal oxide nanoparticles for potential photocatalytic activity.

Zinc oxide nanoparticles have amazing biological applications and are included in ointments used for wounds treatment, heartburns and regeneration of bone [41]. Moreover, ZnONPs have exhibited high toxic potentials against different multidrug-resistant human microorganisms and can be recommended as another possible medication [42]. In addition, ZnONPs with their unique optical and catalytic features are designed as photochemical degradation nanomaterials [43].

Considering the curing efficiency of metal oxide nanostructures synthesis, especially ZnONPs using natural biomasses (plant extracts), the objective of the present work is the eco-friendly synthesis and characterization of ZnONPs using oat biomass as a natural reducing agent. The biomedical potential of the prepared ZnONPs was separately investigated for antibacterial, antioxidant and photocatalytic properties.

## 2. Results and Discussion

### 2.1. Green Synthesized ZnONPs Characterization

**Figure 1:**
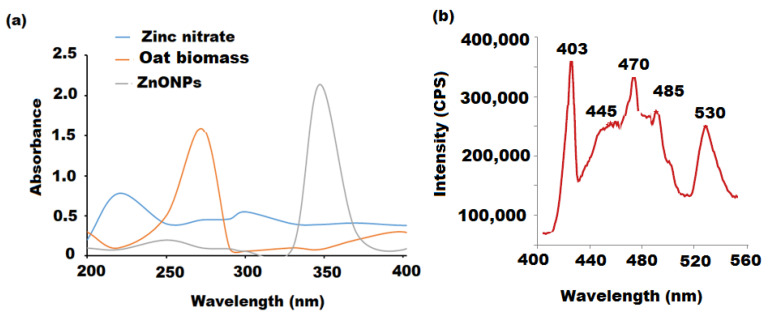
(**a**) UV-Vis spectra of green synthesized ZnONPs using oat biomass. The recorded spectra screened in the wavelength range 200–400 nm. (**b**) Photoluminescence spectrum of green synthesized ZnONPs using oat biomass.

The formation of ZnONPs was characterized using various spectroscopic methods. The optical properties of the as-prepared ZnONPs were detected using UV-vis detection. The absorption spectra showed a clear broad absorption peak at 355 nm for ZnONPs (Figure 1a). The change in the color of oat biomass from yellowish to pale white after boiling for 20 min, then to white after heating at 500 °C for 2 h, might be attributed to the photoexcitation of surface plasmon vibrations from the valence to the conduction band of the formed ZnONPs [44]. These color changes revealed the complete interaction of oat biomass and zinc nitrate hexahydrate producing ZnONPs. The band gap of the synthesized ZnONPs was estimated by the following equation:

Eg = *hv* = hc/λ (1)

where h, c and λ are Planck’s constant (6.626 × 10^−34^ J s), the velocity of light (3 × 10^8^ m/s) and the wavelength (nm), respectively. The band gap was found to be 3.61 eV at the wavelength λ_max_ of 355 nm, which confirmed the formation of nanoparticles when compared with the band gap of ZnO bulk (3.3 eV). The optical outcomes of ZnONPs match those previously reported [45]. Moreover, in nanomaterials, the relation between the size diameter of the particles and the band gap is inversely proportional. The size diameter of nanoparticles is contrarily proportional to the corresponding band gap. The increase of nanoparticles, the decrease of band gap, but it never reaches zero [46].

Photoluminescence (PL) detection was used to determine the emission properties of ZnONPs (Figure 1b). The photoluminescence of the ZnONP sample showed five significant emission bands at 403, 445 and 470 nm (blue bands), 485 (green band) and 530 nm (red bands). The three blue bands are correlated to the defect structures in the ZnO crystal. However, the two green and red bands can be corresponded to the transition between the oxygen vacancy and interstitial oxygen [47]. 

**Figure 2:**
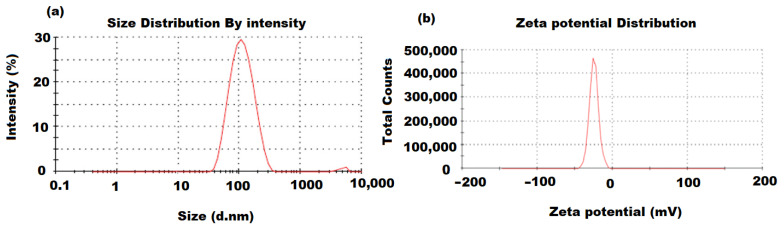
(**a**) Dynamic light-scattering distribution by intensity and (**b**) zeta-potential distribution (mV) of green synthesized ZnONPs using oat biomass.

A dynamic light-scattering analysis was employed to calculate the distribution and average particle size of the green synthesized ZnONPs. The obtained ZnONP particle size was approximately around 100 nm (Figure 2a). A further analysis (zeta potential) was performed to determine the constancy and surface charge of the pre-formed ZnONPs. The recorded results indicate that the moderate stability of the formed ZnONPs resulted from the negatively charged groups of the capping molecules present on the ZnONPs’ surface. The evaluation of the zeta-potential depends on the motion of ZnONPs under the effect of the applied electric field. The determined average value of zeta-potential resulting from the ZnONP analysis was −27.6 mV, revealing the high stability of the prepared ZnONPs (Figure 2b).

**Figure 3:**
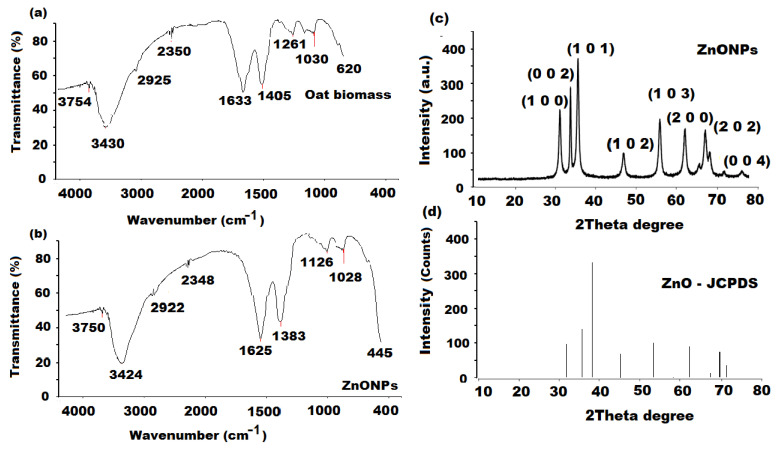
FT-IR spectra of (**a**) oat biomass and (**b**) ZnONPs at wave numbers in the range 4000–400 cm^−1^ and XRD analysis of (**c**) ZnONPs synthesized using oat biomass and (**d**) standard ZnO given by JCPDS (card number: 01-089-1397).

The FT-IR spectra (4000–400 cm^−1^) were recorded to study the possible existence of biomolecules responsible for the reduction and capping of the green synthesized ZnONPs. The particular vibration bands were identified. The FT-IR spectra of oat biomass before and after the reaction of zinc nitrate hexahydrate to ZnONPs are shown in Figure 3a,b. The oat biomass spectra showed various absorption bands at 3754 (medium O-H stretching), 3430 (strong O-H stretching), 2925 (C-H stretching) [48], 2350 (medium N-H amide II), 1633 (strong C=C alkene), 1405 (symmetric stretching of carboxyl side groups of amino acids residue), 1261 (amide III band of protein) [49], 1030 (C-N stretching vibration of amine) and 620 cm^−1^ (strong C-halo, alkyl halide). The previously observed bands were shifted to 3750, 3424, 2922, 2348, 1625, 1383, 1126, 1028 and 445 (Zn-O band) cm^−1^ in the ZnONPs sample [47]. The recorded results confirm that the synthesized ZnONPs were surrounded by the metabolites of biomolecules such as tocopherols and tocotrienols, phenolic acids, carbohydrates and phenolic alkaloids (avenanthramides). The reduction of zinc nitrate into Zn-O can be conducted by the ability of C=O groups of amino acids and proteins to act as reducing and capping to the surface of formed Zn-O. 

An XRD analysis was also performed at ambient degree to evaluate the crystalline structure and phase purity of the green synthesized ZnONPs using λ = 1.5418 Å, 30 mA, 35 kV, 0.02° and 0.3 s/point as K_α_ radiation, operating current, voltage, resolution scan rate, respectively. The XRD pattern displayed various significant peaks at the 31.13° (1 0 0), 34.53° (0 0 2), 37.22° (1 0 1), 47.67° (1 0 2), 57.18° (1 0 3), 63.07° (2 0 0), 68.21° (2 0 2) and 72.03 (0 0 4) orientations (Figure 3c). The diffractogram showed an excellent matching with the hexagonal phase (wurtzite structure) when compared with the results of the Joint Committee on Powder Diffraction Standards (JCPDS; card No. 01-089-1397) (Figure 3d) and no impurity peaks were observed. The average crystallite size of the ZnONP sample was estimated obeying Scherer’s equation:

D = 0.9λ/βCosƟ(2)

where D, k, λ, and β represent the average crystal size, shape factor (0.9), wavelength (0.15416) and Bragg angle Ɵ of the X-ray (1.5406 Å) Cu Ka radiation, respectively. The average ZnONPs crystallite size was calculated as 17.52 nm.

**Figure 4:**
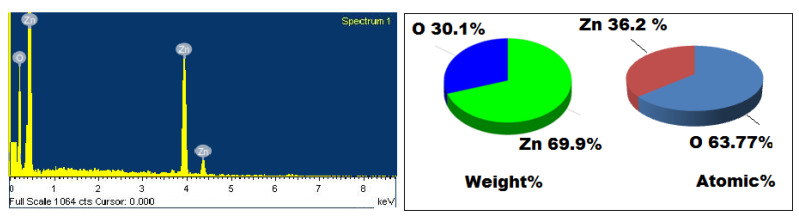
EDX spectrum of ZnONPs synthesized using oat biomass. Weight % of Zn, 69.9%; O, 30.1%. Atomic % of Zn, 36.2%; O, 63.77%.

An EDX detection was applied to quantify the chemical compositions of the biogenic synthesized ZnONPs using oat biomass. The EDX spectrum showed significant signals corresponding to the Zn (weight%, 69.9%; atomic %, 36.2%) and O (weight%, 30.1%; atomic % 63.77%) elements, revealing the formation of ZnONPs (Figure 4). The results revealed the presence of metallic zinc oxide with high purity and no additional peaks corresponding to other elements were recorded.

**Figure 5:**
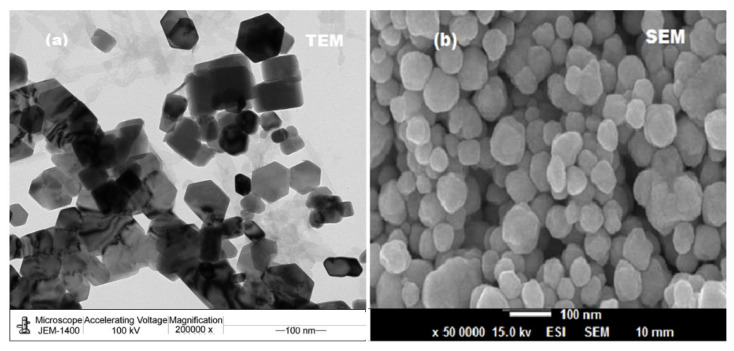
(**a**) TEM and (**b**) SEM images of ZnONPs synthesized using oat biomass.

A microscopic analysis was performed to visualize the surface structure (size andshape) of the synthesized ZnONPs. The size and shape of ZnONPs are demonstrated in Figure 5a. This image revealed that the formed nanoparticles were nanocrystalline hexagonal in shape with a size distribution of around 100 nm. The SEM image demonstrated that the clusters of ZnONPs were nearly hexagonal with rough surface (Figure 5b).

### 2.2. Thermal Stability of Biosynthesized ZnONPs

**Figure 6:**
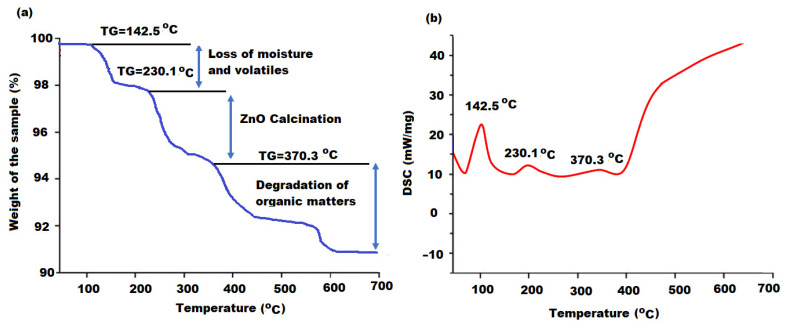
(**a**) Thermogram of ZnONPs at the temperature range 80–700 °C and (**b**) diffraction scanning calorimetry graph of ZnONPs synthesized with oat biomass extract.

The thermal behavior of the biosynthesized ZnONPs using oat biomass extract was investigated through the TGA/DSC mode. As the synthesis of ZnONPs was carried out at 80 °C, the effect of temperature variation on the stability of the formed metal oxide nanoparticles was examined [50]. Figure 6a displays the thermogram behavior of ZnONPs in the temperature range 80–700 °C. The obtained results show that the nominal overall loss of biosynthesized ZnONPs was around 9.5% indicating the significant thermal stability of the sample. Moreover, it was reported that the zinc nitrate precursor that had been used in the synthesis process was completely converted to thermal stable ZnONPs [51]. The thermogram displayed three remarkable regions of weight losses and diffraction scanning calorimetry (DSC) was applied to interpret these regions (Figure 6b). 

The recorded peaks that appeared at 140.5, 230.1 and 370.3 °C were related to the loss of moisture and volatile components from particles surface (2.1 w% loss), conversion of Zn(OH)_2_ to complete calcined ZnONPs (4.5 w% loss) and production of ZnONPs from the complete degradation of organic matters (2.9 w% loss). No other peak was detected at 700 °C, proving the low-temperature calcination of ZnONPs at 400 °C. According to the obtained results, it can be concluded that the bioactive compounds in the oat biomass extract mainly had reduction abilities, with a minimum chelation interaction. The reduction of zinc nitrate to Zn^2+^ ions by the bioactive components was performed in the first stage of synthesis with low chelation of a Zn-bioactive component complex. These bio-complex products were further degraded to ZnO by a further oxidation process of Zn^2+^ to ZnO to establish the proper formation of metallic ZnONPs from zinc nitrate.

### 2.3. Antibacterial Activity

ZnONPs have been reported for the advancement of next-generation nano-antibiotics against various pathogens to avoid multidrug resistance [52,53]. These metal oxide nanostructures exhibit extraordinary physicochemical features, such as (crystallinity, porosity, particle size, and shape) [54]. According to these properties, ZnONPs possess an immense antimicrobial potential versus several pathogens, including Bacillus subtilis, Staphylococcus aureus, Escherichia coli, and Pseudomonas aeruginosa [55,56,57,58].

**Figure 7:**
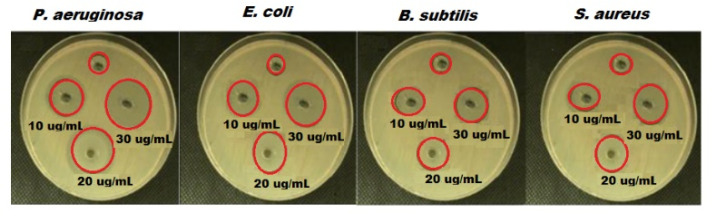
Antibacterial activity of different concentrations of green synthesized ZnONPs using oat biomass against four bacterial strains; the positive control was 25 µg mL^−1^ of tetracycline and the negative control was Milli-Q water.

The antibacterial effect of green synthesized ZnONPs with oat biomass was studied and the outcomes confirm that ZnONPs exhibited excellent dose-dependent manner antibacterial potential (Table 1). The calculated inhibition zones were found as *E. coli* (16 mm), *P. aeruginosa* (17 mm), *S. aureus* (12 mm) and *B. subtilis* (11 mm) for ZnONPs (Figure 7). Thus, the recorded results reveal that the ZnONPs synthesized by oat biomass showed excellent antibacterial potential against all bacterial strains. The highest activity was noticed versus *P. aeruginosa* and *E. coli* using 30 µg mL^−1^. Moreover, the high antibacterial behavior of ZnONPs (oat biomass) could be ascribed to their minimum particle size and shape, and the bioactive feature of oat phytochemical components such as vitamin E, sterols, phytic acid, carotenoids, β-glucan and phenolics [59]. 

#### 2.3.1. Bacteriostatic and Bactericidal Estimation

The bacteriostatic (MIC) and bactericidal (MBC) values of ZnONPs against *P. aeruginosa* and *E. coli* were studied using the agar well diffusion method. The MIC is known as the minimum nanoparticle concentration that inhibits bacterial growth [60]. In the current study, the suitable least concentration to inhibit the growth visibility of *P. aeruginosa* and *E. coli* was estimated after 24 h incubation time at 37 °C. It was observed that the gradual increase in ZnONP concentration from 5 to 640 µg mL^−1^ caused a remarkable reduction in the viability of bacterial cells (*p* < 0.05). The measured MIC was 160 µg mL^−1^ for *P. aeruginosa* and *E. coli*. The size of ZnONPs and their concentrations play a crucial role in antibacterial potential. As previously reported, antibacterial activity of ZnONPs has been changed with respect to the size and concentration-dependent manner [61]. The large surface area of the ZnONPs, due to their small size, enhances their penetration of the microorganism cell through the cell membrane and improve the antibacterial effect with their high concentration [62]. 

**Figure 8:**
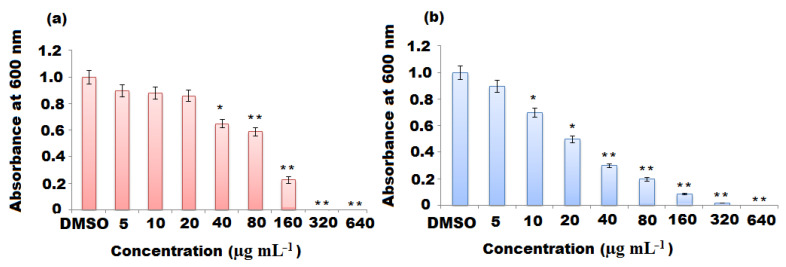
Minimum bactericidal concentration (μg mL^−1^) of the green synthesized ZnONPs using oat biomass against (**a**) *P. aeruoginosa* and (**b**) *E. coli*. Results are expressed as mean ± SD after triplicate studies. The * and ** in the bars represent S.E. (* *p* < 0.05, ** *p*< 0.01).

**Figure 9:**
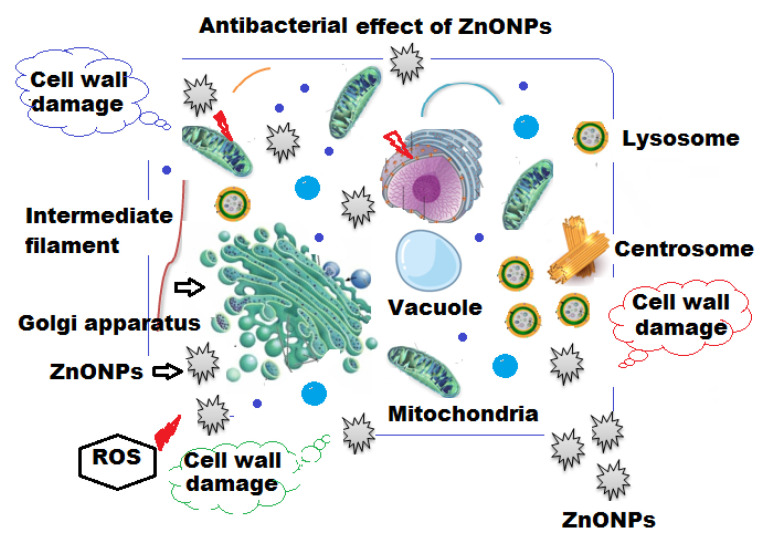
The possible mechanism of antibacterial effect of green synthesized ZnONPs using oat biomass against the bacterial cell.

MBC is known as the least concentration of test sample compound that can lead to pathogenic cell death under a particular condition through a fixed time period (Figure 8a,b). The MBC for *P. aeruginosa* and *E. coli* were 160 and 320 μg mL^−1^ of ZnONPs, respectively (Table 2). Based on the unique physical and chemical properties of ZnONPs, different possible mechanisms of their antibacterial effects have been suggested, with specific interactions such as adsorption, release of Zn^2+^ ions, generation of reactive oxidative species (ROS) [63,64] and intercellular responses of microbial cells, such as lipid peroxidation, cell membrane damage, energy metabolism inhibition and disruption in DNA replication [65,66]. As can be seen from Figure 9, it was demonstrated that the positively charged ZnONPs interacted with the negatively charged cell wall of bacterial cells and, after adsorption, they internalized into the bacterial cell causing loss of cell integrity, rupture of the cell membrane and further oxidative stress due to lipid peroxidation leading to generation of ROS, damage of DNA and inhibition of the bacterial growth.

#### 2.3.2. Morphological Changes under SEM

**Figure 10:**
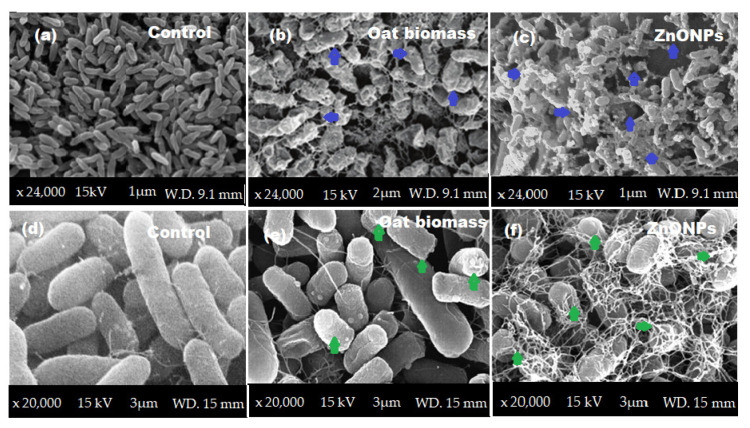
SEM images using 15 kV and magnifications ×24,000 and ×20,000, (A) *P. aeruoginosa* and (B) *E. coli*: (**a**,**d**) control; (**b**,**e**) cells treated with oat biomass; and (**c**,**f**) cells treated with ZnONPs and changes in shape.

The morphological changes in the *P. aeruginosa* and *E. coli* surfaces were investigated under SEM. As a result, the cells treated with oat biomass were swallowed, with a slight change in the morphological shape (Figure 10b,e). The surface coating of bacterial cells by ZnONPs showed significant changes in the shape of bacterial cells with significant cell damage (Figure 10c,f). ZnONPs penetrated the peptidoglycan membrane of *P. aeruginosa* and *E. coli*, causing cell membrane damage, releasing the contents of bacterial cell and, resulting in cell death. These results were compared with the control (untreated bacterial cells) (Figure 10a,d).

### 2.4. Antibacterial Activity of ZnONPs Using Various Plant Biomasses

A comparative study was performed to evaluate the antibacterial activity of ZnONPs prepared by oat biomass and the previously biosynthesized ZnONPs using different plant parts, such as seeds, fruits, leaves, stems and roots. The use of natural extracts of plants produces extensive phytochemical compounds that are cheap, eco-friendly and provides highly purified nanoparticles [67]. Plant extracts are the most preferred natural source for the biosynthesis of nanoparticles with various shapes, particle sizes and stability [68]. They reduce the metal of metal oxides into zero valences with the aid of phytochemicals, including vitamins, amino acids, phenolic compounds, polysaccharides, proteins, alkaloids, terpenoids and sterols, that are secreted from the plants [69]. Table 3 shows the plant extract-mediated synthesis of ZnONPs [70,71,72,73,74]. In the past decades, studies have reported that the green synthesized ZnONPs have an important role in industrial products such as coating, cosmetics, paint, plastics and rubber. Recently, biosynthesized ZnONPs have played a crucial role in biomedical aspects, particularly in the anticancer and antipathogenic fields. These are due to their potential capability to produce excess free reactive oxidative species (ROS), release zinc ions and cause cell damage. Additionally, ZnONPs have also been potentially suggested for antidiabetic treatment due to their ability to keep the structural integrity of insulin. Moreover, ZnONPs have exhibited excellent photoluminescent characteristics and have served as some of the main effective metal oxides in bioimaging. Therefore, biosynthesized ZnONPs can be considered as among the main safe metal oxides used in progressing biomedical applications [75]. 

### 2.5. Antioxidant Potential of ZnONPs

The DPPH assay is commonly applied to evaluate the antioxidant potential due to its sensitivity to determine low concentrations of active components [76]. It is a nitrogen-centered free radical; therefore, any substance that scavenges remarkable amounts of DPPH could reduce the quantities of other reactive nitrogen species in living cells. The influence of several concentrations of ZnONPs and oat biomass on DPPH’ free radical antioxidant potential is presented in Table 4. The data demonstrated that both ZnONPs and oat biomass have free radical scavenging activity. However, the synthesized ZnONPs exhibited stronger scavenging activity against DPPH than oat biomass. The DPPH potentials of ZnONPs and oat biomass were observed to elevate in a dose-dependent manner. At the concentrations of 25–100 μg mL^−1^, the green synthesized ZnONPs exhibited scavenging potential from 58 to 67% with an average IC_50_ value of 0.88 ± 0.03. The antioxidant activity was lower than that of ascorbic acid at 100 μg mL^−1^ (73%). For the ABTS assay, the pre-formed radical monocation of 2,2′-azinobis-(3-ethylbenzothiazoline-6-sulfonic acid) (ABTS*^+^) resulted from the oxidation of ABTS with potassium persulphate. It was reduced by the effect of hydrogen-donating antioxidant materials [77]. The ability of ZnONPs to reduce ABTS to ABTS^+^ ions was also notably higher than that of oat biomass at different concentrations, from 25 to 100 μg mL^−1^, by 53–71% (IC_50_ value of 0.73 ± 0.05). The outcomes of this study reveal that oat biomass is an excellent natural source of phenolic compounds, which are important secondary metabolites responsible for antioxidant activity. In addition, the tunable physical and chemical properties of ZnONPs showed significant antioxidant activity.

### 2.6. Photocatalytic Influence of ZnONPs

**Figure 11:**
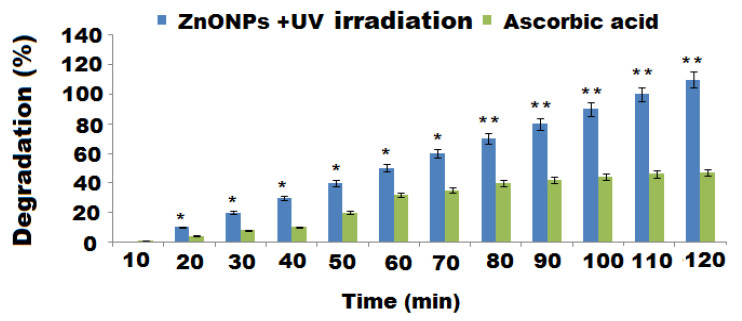
The photocatalytic effect of ZnONPs calculated as % of RB dye degradation in the presence of UV irradiation with respect to ascorbic acid as control. * and **, *p* < 0.05.

The photocatalytic influence of the pre-prepared ZnONPs to produce RB degradation was investigated in an aqueous solution under visible irradiation. The decomposition of RB was determined by measuring the intensity of the absorption peak maxima at 550 nm against the irradiation time. The photodecomposition of RB dye enhanced with time and the complete degradation was almost achieved (98%) with decolorization after 120 min (Figure 11). The outcomes of the current study provide an excellent catalytic behavior of the green formed ZnONPs towards the reductive decomposition of RB dye and the outcomes are matched those of previously addressed studies [78]. A linear relationship (Beer–Lambert law) was obtained from plotting the concentrations of RB against the absorption maxima at 550 nm. This could be ascribed to the stable number of photons available for photodecomposition. The capping layer of the reducing agent (oat biomass) on the ZnONPs’ surface may have also enhanced the potential adsorption between ZnONPs and RB molecules. Consequently, the redox reaction between RB and the reducing agent can be displayed more rapidly for nanoscale particles [79]. The obtained results reveal that the high reactivity of the large surface area of ZnONPs made them efficient photocatalysts for dye degradation under UV light. The possible reduction–oxidation mechanism can be represented as follow:

RB/ZnO + *hv* RB (visible light) → RB*/ZnO (3)

RB*/ZnO → RB^+^/ZnO + e^−^ (4)

e^−^ + O_2_ → O_2_^−^ (5)

H_2_O + O_2_^−^ → °OOH + OH^−^ (6)

2 °OOH + O_2_ → H_2_O (7)

°OOH + H_2_O + e^−^ → H_2_O_2_ + OH^−^ (8)

H_2_O_2_ + e^−^ → °OH + OH^−^ (9)

H_2_O_2_ + °O_2_^−^ → °OH + OH^−^ + O_2_ (10)

The generation of electron–hole pairs, resulting from the valance-conduction band transfer of excited electrons, explains the mechanism of photocatalysis in dye under irradiation. The dye molecules were oxidized to non-toxic products (water, carbon dioxide, etc.) due to the formation of hydroxyl radicals. The high stability, conductivity and unique optical features of ZnONPs enable suitable trapping of photoexcited electrons on their surface and prevent electron–hole recombination [80]. Moreover, the photocatalysis of ZnONPs under visible light could be produced due to surface plasmon resonance, the generation of free radicals and the interaction with oxygen molecules resulting from the collective oscillations of electrons. Additionally, the formation of positive holes conducted from electron excitation resulted in the degradation of RB dye [81].

## 3. Materials and Methods

### 3.1. Collection of Oat Biomass

After oat seed harvest, oat biomass was collected and cleaned by washing thoroughly with tap water, then dried at 80 °C for 12 days to eliminate the residual moisture. The obtained oat biomass was further grinded using an electric grinder (Vermeer, model HG-200) and sieved using a stainless-steel fine-mesh sieve to obtain a homogenous powder.

### 3.2. Materials and Solvents

Zinc nitrate hexahydrate (98%), dimethylsulfoxide (DMSO, 96%), ascorbic acid (99%), osmium tetroxide (99.8%), glutaraldhyde (50% in water), phosphate-buffered saline (pH 7.4), ethanol (98%), methanol (99.85%), potassium persulfate (98.5%), 2,2-diphenyl-1-picrylhydrazyl (DPPH, 95%) and 2,20 -azino-bis [3-ethyl benzo thiazoline-6-sulphonic acid] (ABST, 98%) were purchased from Sigma-Aldrich (Hamburg, Germany).

### 3.3. Bacterial Strains and Nutritional Matrices

All bacterial strains included in this study were provided by Microbiology Department, King Saud University, Saudi Arabia. The *Bacillus subtilis* (ATCC 6633), *Escherichia coli* (ATCC 25966), *Staphylococcus aureus* (ATCC 25923), *Pseudomonas aeruginosa* (ATCC 27853) bacterial strains were used. The pre-culture of these isolates was performed using nutrient agar (Oxoid).

### 3.4. Preparation of Aqueous Oat Biomass Extract

The aqueous extract of oat biomass was prepared by adding 20 g of dried oat powder to 200 mL of boiled distilled water at 100 °C for 1 h. After cooling at room temperature, the obtained extract was purified using filter paper (Whatman filter paper No. 40) and kept in a refrigerator at 4 °C.

### 3.5. Preparation of ZnONPs Using Oat Biomass Extract

The green biosynthesis of ZnONPs was based on reducing, capping and stabilizing zinc nitrate hexahydrate precursor using the phytochemical components of oat extract (pale, yellowish color), which contains bioactive compounds, including *β*-glucan, phenolic acids (ferulic, p-coumaric, caffeic, vanillic and hydroxybenzoic acid), phenolic alkaloids (avenanthramides), vitamin E (α-Tocotrienols and α-tocopherols), starch and protein [82]. These bioactive compounds have various functional groups (-OH, COOH, -NH, C=O, -SH) which serve as natural reducing, capping and stabilizing agents for nanoparticle synthesis [83]. The biosynthesis process was conducted by heating oat extract (50 mL) to 80 °C using a magnetic stirrer heater. Approximately, 5.0 g of zinc nitrate hexahydrate was mixed with the heated extract under stable magnetic stirring at 80 °C for 20 min until the formation of light, yellow-colored ZnONPs. The formed nanoparticles were centrifuged at 1500 rpm for 5 min and filtrated using filter paper (Whatman No 1). The filtrate was washed 3 times using deionized water and collected in a ceramic crucible, then calcined in a muffle oven for 2 h at 400 °C and stored for further use at 4 °C [84]. 

### 3.6. Spectroscopic and Microscopic Characterization

The UV-Vis spectra were recorded using a spectrophotometer (Shimadzu Corporation, Kyoto, Japan) at an absorption wavelength range of 200–400 nm for the primary characterization of the synthesized ZnONPs. Fourier transform infrared spectroscopy (FTIR; PerkinElmer, Waltham, MA, USA) was performed in the IR region of 4000–400 cm^−1^ to study and confirm the possible predictable functional groups. X-ray diffraction (XRD; Shimadzu XRD-6000 diffractometer; Kyoto, Japan) was also carried out to study the crystalline shape of the synthesized ZnONPs using Kα radiation (*λ* = 1.5418 Å; operating current, 30 mA, voltage, 35 kV) at scan rate of 0.3 s/ point and a 0.02° resolution at room temperature. The surface morphology of the prepared ZnONPs was examined under a scanning electron microscope (SEM; JSM-7610F; JEOL, Tokyo, Japan) and a transmission electron microscope (TEM; JEM-2100F; JEOL Ltd., Tokyo, Japan). Furthermore, the elemental content of the synthesized ZnONPs was determined using energy-dispersive X-ray spectroscopy (EDX; JSM-7610F; JEOL, Tokyo, Japan) in connection with an SEM microscope. Thermal stability of the biosynthesized ZnONPs was evaluated using a thermogravimetry and differential scanning calorimetry (TGA/DSC) analyzer (TGA/DSC-Seiko Exstar 6300, Tokyo, Japan) in the temperature range of 80–700 °C.

### 3.7. Antibacterial Activity

The antibacterial potential of green synthesized ZnONPs with oat biomass was screened in terms of zone of inhibition using an agar well diffusion assay [85] against different bacterial Gram-negative (*Escherichia coli* ATCC 25966 and *Pseudomonas aeruginosa* ATCC 27853) and Gram-positive (*Staphylococcus aureus* ATCC 25923 and *Bacillus subtilis* ATCC 6633) strains. The nutrient agar (Oxoid) was used to pre-culture of bacterial stains. The bacterial suspension of 0.5 McFarland turbidity of each stain was prepared in 5 mL nutrient broth tubes for the antibacterial study. Sterile cotton swabs were used to load the resulted bacterial suspensions on the surface of Mueller Hinton (Oxoid) plates. Wells (6 mm) were assembled on the agar plates surface using a sterile cork borer and then 100 μL of each ZnONPs (10–30 μgmL^−1^ DMSO) was loaded. The loaded plates were aerobically kept for 24 h at 37 °C.

### 3.8. Bacteriostatic and Bactericidal Estimation

In the present assay, the minimum inhibitory concentration (MIC) of ZnONPs against *P. aeruginosa* and *E. coli* and was estimated (three times) by applying the micro-broth dilution method. The investigated concentrations were taken in the range from 5 to 640 μg mL^−1^. This assay was conducted by performing 2-fold serial dilutions in 96-well plates. The positive control (bacterial cells and broth) was prepared in the first column. The last column (Milli-Q water) was considered as the negative control.

Each bacterial suspension was spreading and after the incubation time (24 h at 37°C), ELISA reader (Biotech, Wuxi, China) applied to read out the results at 600 nm. For matching the results, tetracycline (TE; 25 μg mL^−1^) and DMSO were used as positive and negative controls, respectively. The least concentration of ZnONPs which could lead to a complete bactericidal effect (MBC) was determined by loading amounts of first turbid samples with no visible bacterial growth. The treated samples were carefully distributed on plates containing nutrient agar using a sterile L rod and incubated for 12 h at 37 °C.

### 3.9. Microscopic Study of P. aeruginosa and E. coli

The surface morphology of both treated and untreated *P. aeruginosa* and *E. coli* was investigated under SEM to evaluate the effect of ZnONPs. Prior to examination under a microscope, the treated *P. aeruginosa* and *E. coli* bacteria pieces (5-by-10 mm) were cut and kept in glutaraldehyde in a phosphate-buffered saline (3%) solution for 1 h. Then, they were fixed in an osmium tetroxide (2%) solution for 1 h. Ethanol and CO_2_, were utilized for tissues dehydration. The dried tissues were fixed on Al stubs with Ag pain vacuum coated with Au–palladium alloy and investigated using SEM at a 15 kV acceleration voltage.

### 3.10. Antioxidants

The antioxidant potential of ZnONPs was determined using two different radical scavenging assays, 2,2-diphenyl-1-picrylhydrazyl (DPPH) and ABTS (2,20 -azino-bis [3-ethyl benzo thiazoline-6-sulphonic acid]). The DPPH free radical scavenging potential of oat and ZnONPs was evaluated following standard methods [86]. Prior to the investigation, 1.0 × 10^−4^ mol L^−1^ DPPH, three different concentrations (25, 50, 100 μg mL^−1^) of oat and ZnONPs and ascorbic acid (AA) were prepared in menthol. Approximately, 50 μL of DPPH solution was mixed with different concentrations of oat and ZnONPs, as well as AA, in a 96-well microplate. The prepared mixture was continuously shacked in a dark place for 30 min using an orbital shaker. The absorbance of the sample was determined using a UV-Vis BioTek microplate (Biotech ELx 800; Biotek instruments, Inc, Highland Park, CA, United States) after incubation for 30 min at 518 nm against methanol as blank.

The scavenging ability was calculated using the following equation:

% DPPH free radical scavenging effect = C_r_ − T_r_/C_r_ × 100 (11)

where Cr and Tr represent the absorbance of the control and test samples, respectively.

The 2, 20 -azino-bis [3-ethyl benzo thiazoline-6-sulphonic acid]) (ABTS) free radical scavenging assay was also used to evaluate the antioxidant activity of oat extract and ZnONPs using a reported standard method [87]. Briefly, 7.4 × 10^−3^ ABTS and 2.6 × 10^−3^ mol L^−1^ of potassium persulfate solutions were separately prepared. The working solution of ABTS was obtained by mixing equal amounts of the previously prepared stock ABTS and potassium persulfate solutions and kept without stirring for 12 h in a dark place. The analysis was conducted by mixing 125 μL of the ABTS working solution with 10 μL of different concentrations (25, 50, 100 μg mL^−1^) of oat and ZnONP tested samples, as well as ascorbic acid as a control. The final solution was left for 2 h in a dark place. The absorbance of the samples was recorded at 518 nm using a UV-Vis BioTek microplate vs. methanol as the blank reference. The scavenging potential was calculated using the following equation:

% ABTS free radical scavenging effect = C_r_ − T_r_/C_r_ × 100 (12)

where Cr and Tr represent the control and test samples absorbance, respectively.

### 3.11. Photocatalysis

To investigate the photocatalytic potential of the green synthesized ZnONPs, light irradiation using a UV lamp (Hamamatsu LC8; lamp power of 0.1 W) was utilized to depredate Rhodamine B (RB) dye at a wavelength of 550 nm at ambient temperature. Approximately, 1.0 mg of ZnONPs was mixed with 2.0 mL of RB dye solution (5.0 × 10^−6^ mol L^−1^) and sonicated for 5.0 min, then kept under constant magnetic stirring for 30 min to cofirm an adsorption/desorption equilibrium on the ZnONP surface prior to irradiation. Under the same experimental conditions, a sample free from the nanoparticles was used as a control. In addition, the physisorption of ZnONP surface was evaluated by maintaining the sample in the dark. The detection was conducted by monitoring the samples at intervals of 10 min. Briefly, the collected samples were centrifuged at 8000 rpm for 10 min and the clear layer of each sample was measured using a UV-Vis spectrophotometer. The rate of degradation was detected at λ_max_ 550 nm by measuring the reduction in absorption intensity of RB dye. The following equation was used to estimate the decomposition efficiency (DE):

DE% = A_0_ − A A_0_ × 100 (13)

where A_0_ and A are the initial absorption and absorption intensity after photocatalytic degradation at λ_max_ 550 nm, respectively.

## 4. Conclusions

The present study suggests a clean and simple, safe and cost-effective approach for the green synthesis of ZnONPs using oat biomass. The resulted metal oxide nanoparticles were characterized using several spectroscopic and microscopic analysis to prove the formation of ZnONPs. The results reveal the preparation of ZnONPs with a particle size of around 100 nm. The resulting ZnONPs were screened for antibacterial activity and they exhibited strong antibacterial potential versus different types of bacterial strains. The green synthesized ZnONPs showed significant antioxidant activity. The DPPH potential of ZnONPs showed scavenging effect from 58 to 67%, with an average IC_50_ value of 0.88 ± 0.03 and, in the ABTS method, the ability of ZnONPs to reduce ABTS to ABTS^+^ ions was from 53 to 71% (IC_50_ value 0.73 ± 0.05). In addition, ZnONPs exhibited the highest photocatalytic activity for RB dye degradation (98%) under visible light irradiation for 120 min.

## Figures and Tables

**Table 1:** Antibacterial effect of ZnONPs synthesized using oat biomass on four different bacterial strains.

Bacterial Strains	Inhibition Zone (mm) at Different Concentrations			Positive Control Tetracycline 25 µg mL^−1^
	ZnONPs (Oat Biomass)			
	10 µg mL^−1^	20 µg mL^−1^	30 µg mL^−1^	
*P. aeruginosa*	14	15	17	17
*E. coli*	13	16	16	16
*B. subtilis*	8	11	11	13
*S. aureus*	10	10	12	18
Negative control Milli-Q water	0.0	0.0	0.0	0.0

**Table 2:** Least bactericidal potential of ZnONPs synthesized using oat biomass on *P. aeruginosa*, *E. coli*, *B. subtilis* and *S. aureus*.

Sample Concentration (µg mL^−1^)	CFU mL^−1^			
	*P. aeruoginosa*	*E. coli*	*B. subtilis*	*S. aureus*
Control	TNTC *	TNTC *	TNTC *	TNTC *
5	TNTC *	TNTC *	TNTC *	TNTC *
10	TNTC *	TNTC *	TNTC *	TNTC *
20	TNTC *	TNTC *	TNTC *	TNTC *
40	TNTC *	TNTC *	TNTC *	TNTC *
80	TNTC *	TNTC *	TNTC *	TNTC *
160	2 × 10^2^	3 × 10^3^	5 × 10^5^	4 × 10^4^
320	NIL **	2 × 10^2^	245	135
640	NIL **	NIL **	5	2

* TNTC indicates “too numerous to count”. ** NIL indicates “not completely free from microorganisms <1 CFU mL^−1^”.

**Table 3:** Comparative study of biosynthesis of ZnONPs using various plant biomasses.

Serial. No.	Plant Name	Extraction Part	Size of ZnONPs XRD (nm)	Particle Shape	Phytochemicals (Functional Groups)	Type of Pathogens	MIC (μg mL^−1^)	Ref.
**1**	*Azadirachta indica*	Leaves	18	Spherical	-OH, C=O, -NH, -COOH	*Staphylococcusaureus,* *Pseudomonas aeruginosa, Bacillus subtilis, Proteus mirabilis Escherichia coli, Candida albicans, Candida tropicalis*	6.25–50	[70]
**2**	*Nephelium lappaceum*	Fruit peels	50.95	Needle shape	-OH, H-O-H	*Staphylococcus aureus, Escherichia coli*	—	[71]
**3**	*Pongamia pinnata*	Leaves	26	Spherical, hexagonal, nanorods	-OH, C=O, -C-O-H, -COOH	*Staphylococcus aureus, Escherichia coli.*	100	[72]
**4**	*Berberis aristata*	Leaves	5–25	Needle shape	-OH, -COOH, -NH, -C-H, -C=C	*Bacillus subtilis, Serratia marcescens, Staphylococcus aureus, Escherichia coli, Salmonella Typhi, Klebsiella pneumonia, Bacillus cereus*	64–256	[73]
**5**	*Lawsonia inermis*	Leaves	33	hexagonal	C=O, -OH, -COOH	*Escherichia Coli, Pseudomonas aeruginosa, Staphylococcus aureus, Bacillus subtilis*	100–200	[74]
**7**	*Avena Sativa* (Oat)	Seeds	17.52	Hexagonal	-OH, -COOH, Phenolic ring, -NH, C=O	*Escherichia Coli, Pseudomonas aeruginosa, Staphylococcus aureus, Bacillus subtilis*	160	Current study

**Table 4:** Data obtained from DPPH and ABTS scavenging activity of oat biomass and ZnONPs in comparison with ascorbic acid as control.

Samples	DPPH Radical Scavenging Effect			ABTS Radical Scavenging Effect		
	Conc. (µg mL^−1^)	Scavenging (%)	IC_50_ (µg mL^−1^)	Conc. (µg mL^−1^)	Scavenging (%)	IC_50_ (µg mL^−1^)
Oat biomass	25 75 100	26.37 ± 0.67 32.92 ± 0.14 53.64 ± 0.25	1.65 ± 0.05	25 75 100	35.12 ± 0.24 45.36 ± 0.38 45.36 ± 0.47	1.63 ± 0.07
ZnONPs	25 75 100	58.37 ± 0.67 62.18 ± 0.18 67.64 ± 0.12	0.88 ± 0.03	25 75 100	53.14 ± 0.53 59.36 ± 0.25 71.56 ± 0.84	0.73 ± 0.05
Ascorbic acid	25 75 100	51.36 ± 0.42 56.85 ± 0.73 73.00 ± 0.35	0.35 ± 0.07	25 75 100	52.62 ± 0.58 57.36 ± 0.32 72.54 ± 0.83	0.27 ± 0.02

## Data Availability

The data used to support the findings of this study are included within the article.

## Sample Availability

Samples of the compounds are not available from the authors.
